# Use of First-Line Immune Checkpoint Inhibitors and Association With Overall Survival Among Patients With Metastatic Melanoma in the Anti–PD-1 Era

**DOI:** 10.1001/jamanetworkopen.2022.25459

**Published:** 2022-08-25

**Authors:** Nayan Lamba, Patrick A. Ott, J. Bryan Iorgulescu

**Affiliations:** 1 Harvard Radiation Oncology Program, Boston, Massachusetts; 2 Department of Medical Oncology, Dana-Farber Cancer Institute, Department of Medicine, Brigham and Women’s Hospital, Harvard Medical School, Boston, Massachusetts; 3 Department of Pathology, Brigham and Women’s Hospital, Harvard Medical School, Boston, Massachusetts

## Abstract

**Importance:**

In 2015, first-line programmed cell death 1 (PD-1) immune checkpoint inhibitors (ICI) were Food and Drug Administration (FDA)-approved and National Comprehensive Cancer Network (NCCN)-recommended for patients with stage IV melanoma. Few studies have assessed the overall survival (OS) and usage rate associated with first-line ICI following 2015.

**Objective:**

To determine the rates of ICI use for metastatic melanoma following FDA approval in 2015 and characterize OS associated with first-line ICI use in this patient population.

**Design, Setting, and Participants:**

In this retrospective, nationwide cohort study, adult patients (≥20 years of age) with newly diagnosed stage IV cutaneous melanoma from 2010 to 2019 were identified using the US National Cancer Database (NCDB). Data were released by NCDB in March 2022 and analyzed in June 2022.

**Interventions:**

Patients were compared based on first-line ICI receipt vs not.

**Main Outcomes and Measures:**

The OS and use of first-line ICI in 2016 to 2019 were assessed using multivariable Cox and logistic regression, respectively. To account for immortal time bias in receiving ICI, landmark time points were used (the 50th and 75th percentile times from diagnosis to ICI initiation).

**Results:**

Among 16 831 patients with stage IV melanoma, 11 435 (67.9%) of patients were male; 116 (0.69%) were Asian or Pacific Islander, 475 (2.82%) were Hispanic, 270 (1.60%) were non-Hispanic Black, 15 711 (93.55%) were non-Hispanic White, and 145 (0.86%) were other race and ethnicity; the median (IQR) age at diagnosis was 64 (54-73) years. First-line immunotherapy use increased from 8.9% (127 of 1429) in 2010 to 38.8% (685 of 1766) in 2015, and 62.5% (1223 of 1958) in 2019. Median OS improved from 7.7 months (95% CI, 7.1-8.6 months) in 2010 to 17.5 months (95% CI, 14.9-19.8 months) in 2018. For patients diagnosed in 2016 or later, OS improved with first-line ICI (median OS using the 78-day landmark: 43.7 months [95% CI, 38.1-49.1 months] vs 16.1 months [95% CI, 13.5-19.3 months] for targeted therapy or chemotherapy; adjusted *P* < .001)—even after adjusting for patient, disease, and treatment factors. Results were similar for the 48-day landmark. This included patients presenting with brain metastases (first-line ICI median OS using the 78-day landmark: 19.9 months [95% CI, 17.2-25.0 months] vs 10.7 months for targeted therapy [95% CI, 9.5-12.3 months], adjusted *P* = .001). First-line ICI use varied by patients’ age, insurance status, zip code–level household income, and treating hospital type.

**Conclusions and Relevance:**

Following anti–PD-1 approval in 2015, first-line ICI was associated with substantial OS improvements for patients with stage IV melanoma, including those with brain metastases. As of 2019, 38% of patients still were not receiving first-line ICI in the US, with use varying by patients’ socioeconomic factors.

## Key Points

**Question:**

Following the US Food and Drug Administration’s approval of PD-1 immune checkpoint inhibitors (ICI) for metastatic melanoma, what were the utilization and overall survival associated with ICI in the first-line setting after 2015?

**Findings:**

In this cohort study of 16 831 patients with metastatic melanoma, first-line immunotherapy use increased from 2010 to 2019, although 38% of patients still were not receiving first-line ICI in 2019. Use of ICI was associated with improvements in survival, even among those with brain metastases.

**Meaning:**

First-line ICI therapy was associated with substantial improvements in overall survival among patients with metastatic melanoma, but rates of use remain suboptimal.

## Teaser

This cohort study examines the usage rates of immune checkpoint inhibitors (ICI) and characterizes overall survival associated with first-line ICI use among patients with metastatic melanoma following FDA approval in 2015.

## Introduction

The emergence of immune checkpoint inhibitors (ICI) and BRAF and MEK inhibitors has transformed the therapy of patients with metastatic melanoma over the last decade. In 2011, the United States Food and Drug Administration (FDA) approved the anti–CTLA-4 antibody ipilimumab for patients with stage IV melanoma, followed by the anti–programmed cell death 1 (anti–PD-1) antibodies nivolumab and pembrolizumab in 2014 for the second and subsequent line treatment. With the introduction of ICIs and BRAF/MEK targeted therapies, we and others have shown that the 5-year overall survival (OS) rate for patients with metastatic melanoma has risen from less than 10% to 30% to 50%.^1,2^

In 2015, based on several landmark Phase 3 clinical trials demonstrating substantial improvements in objective response and progression-free survival with first-line ICI compared with chemotherapy in patients with stage IV melanoma, the National Cancer Comprehensive Network (NCCN) recommended—and the FDA approved—PD-1 ICI as first-line standard-of-care therapy for such patients.^3,4,5,6,7,8,9,10,11^ Currently recommended ICI regimens include anti–PD-1 monotherapy with nivolumab or pembrolizumab and combined anti–PD-l and anti–CTLA-4 therapy with nivolumab/ipilimumab. For patients with *BRAFV600-*variant melanoma, combination BRAF and MEK inhibitor therapy with either dabrafenib and trametinib, vemurafenib and cobimetinib, or encorafenib and orbinimetinib are recommended first-line regimens.^11^

These newly available therapies have been practice changing. Although there have been multiple randomized studies demonstrating the efficacy of ICI for patients with stage IV melanoma, large-scale analyses examining the association of first-line ICI with survival at a national level have been lacking. We and others have previously reported on the survival benefits for patients with stage IV metastatic melanoma presenting after the FDA’s initial approvals for second-line ICI.^2,12^ Here, we build on that work to examine how first-line ICI use following FDA approval may be associated with the OS of patients with stage IV melanoma in the US, including among patients with brain metastases, liver metastases, and high serum lactate dehydrogenase levels. Furthermore, as a secondary aim, we assessed the use patterns of first-line ICI by patient, socioeconomic, and care setting factors.

## Methods

### Data Source and Study Design

This retrospective cohort study was conducted in accordance with the Declaration of Helsinki and was approved by the Mass General Brigham institutional review board. We followed the Strengthening the Reporting of Observational Studies in Epidemiology (STROBE) reporting guideline for cohort studies. This study used the National Cancer Database (NCDB): a hospital-based database that contains information for more than 72% of newly diagnosed cancers in the United States.^13,14^ Patients presenting with cutaneous melanomas (ie, C44.0-C44.9) from 2010 to 2019 were identified from the NCDB cutaneous melanoma site data set, with 2019 representing the most recent year for which data were available. This NCDB 2019 Participant Use File was publicly released in March 2022 and analyzed in June 2022. Inclusion criteria included adults (aged ≥20 years) with American Joint Committee on Cancer stage IV (ie, distant metastases) cutaneous melanoma, who had no prior history of cancer, and the patient’s data were reported from the treating institution. The NCDB groups all first-line immunotherapies and biologic therapies into a single variable. However, given that in 2016 ICI became the principal NCCN-recommended immunotherapy choice for the standard of care of stage IV melanoma, and that interleukin-2- and interferon-alfa-based regimens were largely relegated to the second- or later-line setting, herein immunotherapy coding was defined as having received ICI.^3^ Additionally, the NCDB groups all cytotoxic chemotherapies and targeted therapies (eg, BRAF and MEK inhibitors) into: none, single agent, or multiagent. In this population and timeframe, first-line systemic chemotherapy likely overwhelmingly represented BRAF and MEK inhibitor therapy.

### Statistical Analysis

The primary outcome of interest was the OS associated with first-line ICI, which was measured from the date of diagnosis to date of death (or censored at last follow-up). In addition to the analysis of survival in all patients with stage IV melanoma, additional prespecified subset survival analyses were conducted for historically challenging melanoma subpopulations: patients with brain metastases, liver metastases, or elevated serum lactate dehydrogenase (LDH) levels. The NCDB excludes survival information for patients diagnosed in the most recent year, which for this data set was 2019. Kaplan-Meier methods were used to estimate OS with 95% CIs and the log-rank test was used for unadjusted OS comparison. To help ensure that first-line immunotherapy represented ICI, we restricted our analyses to patients diagnosed in 2016 to 2019. Variables that could potentially confound the association between first-line ICI and OS were adjusted for in multivariable Cox regression with 99% CIs, including: patients’ age at diagnosis, Charlson-Deyo comorbidity index, first-line targeted therapy and/or chemotherapy, first-line radiotherapy, resection of a nonprimary site, and site of metastasis. The pretreatment (or within 6 weeks of diagnosis) LDH was reported for 22.6% of patients herein and was therefore evaluated separately. The secondary outcome of interest was whether any patient, tumor, or care setting variables were associated with receipt of first-line ICI, which was evaluated using multivariable logistic regression. This analysis additionally included patients’ sex, race and ethnicity, year of diagnosis, insurance status, zip code–level household income (reported by NCDB as quartiles), primary tumor site, primary site surgery, Commission on Cancer classification of hospital type, and hospital geographical location, as previously described.^2,15^ As with all variables used in our analysis, the race and ethnicity variables were defined by the National Cancer Database and abstracted by certified cancer registrars from the medical records. Patients with missing data were excluded from multivariable analyses.

To help account for immortal time bias (ie, survival time bias) associated with receipt of ICI in both survival and use analyses, patients were only included if they survived at least until a landmark time point, as we have previously described.^2^ Herein, the landmark time points were specified as the 50th and 75th percentiles of time from diagnosis to initiation of ICI: 48 and 78 days, respectively. For the primary analyses, a 2-sided α level of .05 was used, corrected for the 4 hypotheses being tested with the more conservative 78-day landmark: the OS association with first-line ICI vs non–immunotherapeutic systemic therapy in all patients, patients with metastatic brain cancer, patients with metastatic liver cancer, and patients with high LDH stage IV melanoma (ie, *P* < .0125 was considered significant). For the secondary analysis of first-line ICI use, to reduce the risk of false positives, a 2-tailed *P* < .001 was designated as significant. Statistical analyses were performed with STATA version 17.1 (StataCorp). Data were released by the NCDB in March 2022 and analyzed in June 2022.

## Results

**Figure 1.**
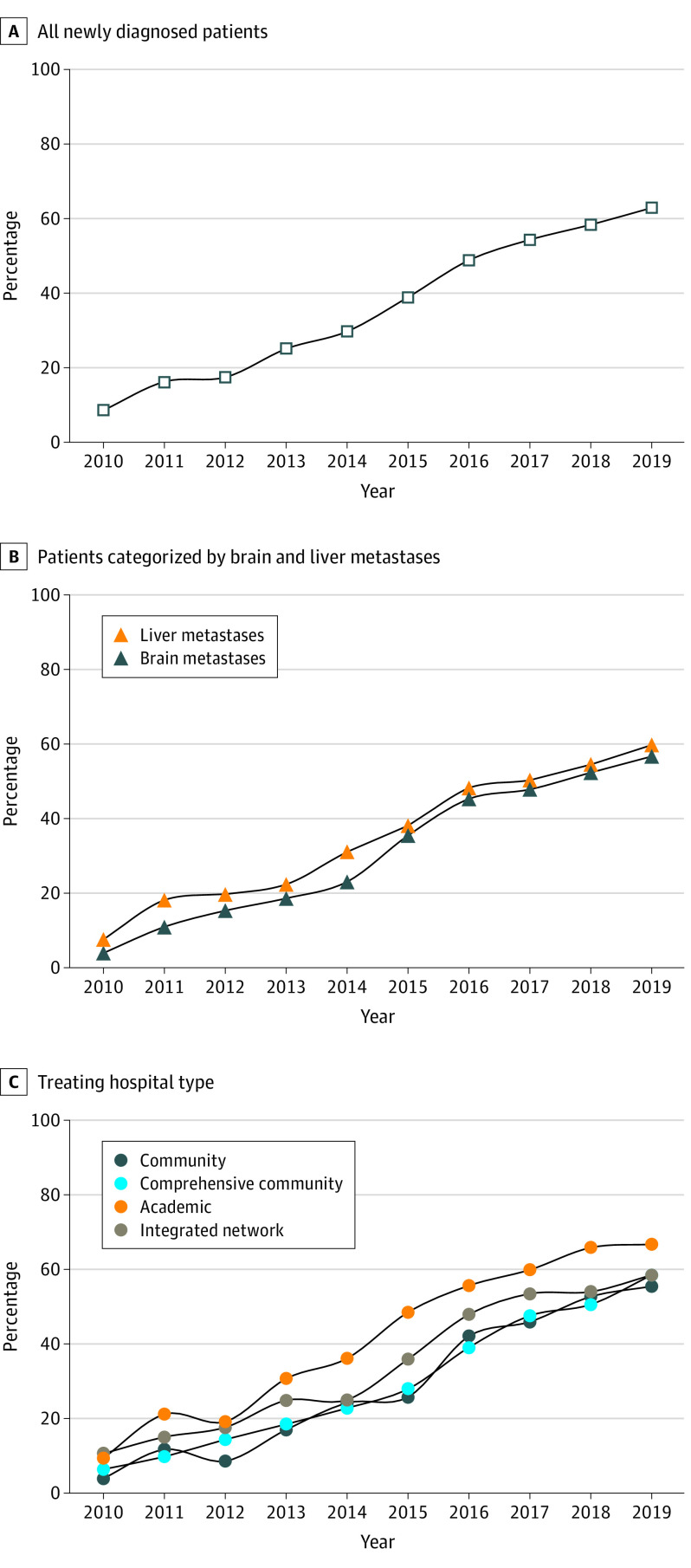
Use of First-Line Immune Checkpoint Inhibitors (ICI) for Patients With Stage IV Melanoma Diagnosed From 2010 to 2019 Percentage of patients with newly diagnosed stage IV melanoma who received ICI in the first-line setting from 2010 to 2019, for (A) all patients, as well as categorized by those with (B) brain and liver metastases and (C) treating hospital type.

Between 2010 and 2019, 16 831 patients presenting with stage IV melanoma met inclusion and exclusion criteria (156 patients were excluded for lacking data about immunotherapy receipt). Among all included patients in this study, 11 435 (67.9%) were male; 116 (0.69%) were Asian or Pacific Islander, 475 (2.82%) were Hispanic, 270 (1.60%) were non-Hispanic Black, 15 711 (93.55%) were non-Hispanic White, and 145 (0.86%) were other race and ethnicity; and the median (IQR) age at diagnosis was 64 (54-73) years. Among these patients, the use of first-line immunotherapy increased from 8.9% (127 of 1429) in 2010 to 38.8% (685 of 1766) in 2015, and to 62.5% (1223 of 1958) in 2019 (Figure 1A-C). Among the patients who did not receive first-line immunotherapy, first-line targeted therapy or chemotherapy was reported in 40.6% (513 of 1265) of patients in 2010, 28.2% (296 of 1049) in 2015, and 25.3% (185 of 731) in 2019—most of which received single agent from 2010 to 2014 and multiagent from 2015 to 2019. The median OS for all patients with stage IV melanoma improved from 7.7 months (95% CI, 7.1-8.6 months) in 2010 to 17.5 months (95% CI, 14.9-19.8 months) in 2018 (the last year for which survival data were available).

### Improved Overall Survival Associated With First-Line Immune Checkpoint Inhibitors

**Figure 2.**
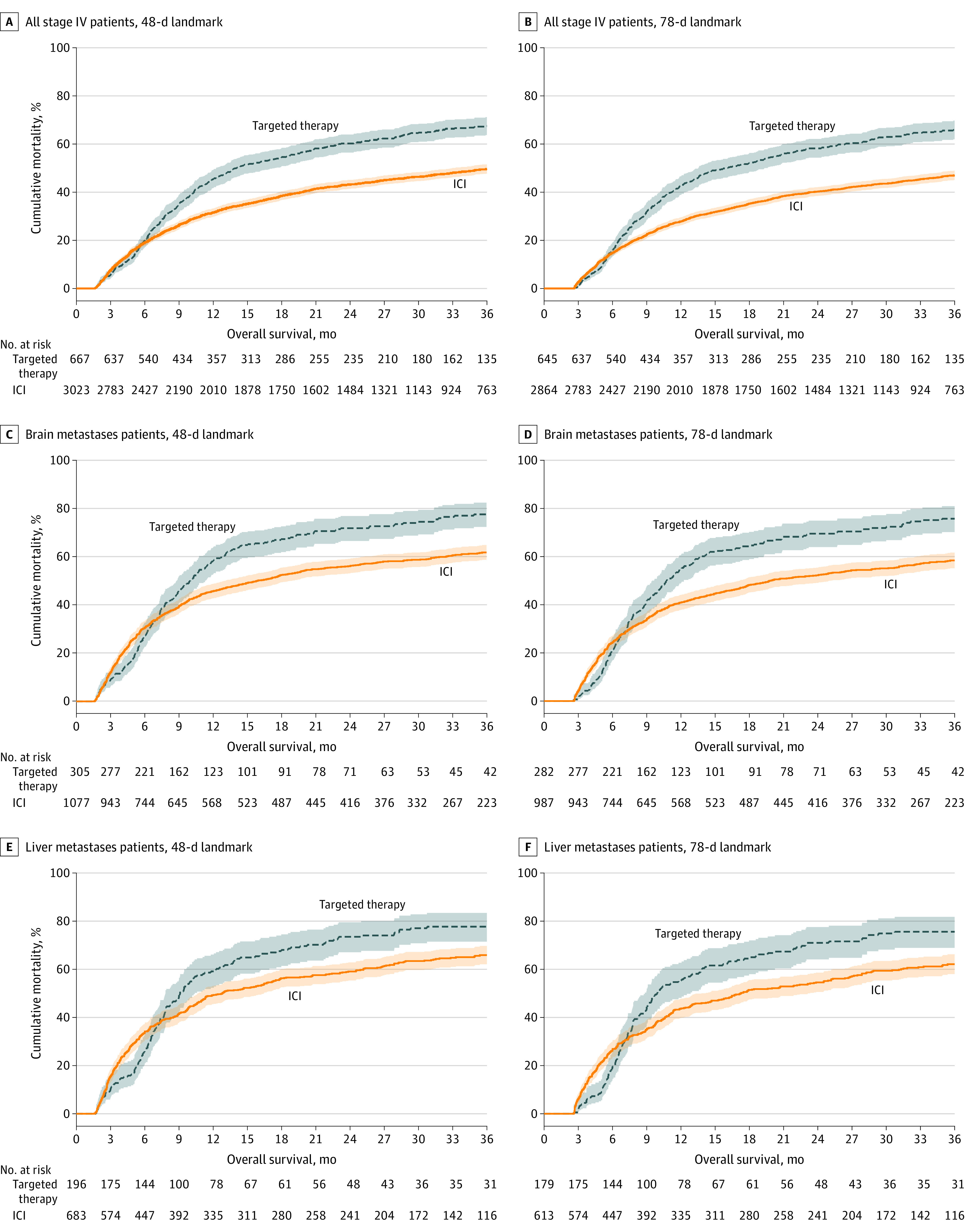
Overall Survival (OS) Associated With First-Line Immune Checkpoint Inhibitor (ICI) Therapy vs Targeted Therapy Among Patients With Stage IV Melanoma Diagnosed Following US Food and Drug Administration (FDA) Approval of First-Line Anti–PD-1 in Late 2015 OS was estimated and plotted using Kaplan-Meier techniques with corresponding 95% CIs for patients that received first-line ICI therapy (orange) and those that only received first-line non-immunotherapeutic systemic therapy (blue). OS was assessed for patients diagnosed in 2016 through 2018. Survival outcomes were not reported for patients diagnosed in the last year of the data set, which was 2019 herein. To account for immortal time bias associated with receipt of ICI, landmark time points were used corresponding to the 50th percentile (ie, 48 days; A, C, E) and 75th percentile (ie, 78 days; B, D, F) of time from diagnosis to ICI initiation.

To evaluate the association between first-line ICI and OS, we restricted our analysis to patients with stage IV melanoma diagnosed in 2016 and onward—when anti–PD-1 was FDA approved for the front-line setting. Median (IQR) follow-up in this population was 13.0 (3.4-32.2) months; 3467 patients (59.9%) died. For patients with stage IV cancer diagnosed in 2016 or later who survived at least until the landmark time points, OS significantly improved with first-line ICI—even after adjusting for patient, disease, and treatment factors, and using either landmark time point (eTable in the Supplement; Figure 2A-B). Even among those patients who survived until the more conservative landmark time point (ie, 78 days), first-line OS was associated with an improved median OS of 43.7 months (95% CI, 38.1-49.1 months; adjusted hazard ratio [HR], 0.72; 99% CI, 0.62-0.85; *P* < .001) compared with 16.1 months (95% CI, 13.5-19.3 months) among patients who received first-line non–immunotherapeutic systemic therapy (ie, likely targeted therapy). First-line ICI also demonstrated durable OS benefits, with a 53.0% 3-year OS rate (95% CI, 51.0%-54.9%) compared with 35.5% for patients with non-immunotherapeutic systemic therapy (95% CI, 30.6%-40.4%) from the 78-day landmark.

### First-Line Immune Checkpoint Inhibitors in Historically Challenging Melanoma Subpopulations

We additionally investigated the association of first-line ICI with the OS in 2 historically challenging contexts: patients with melanoma metastases to the brain and liver. For the 3068 patients (40.2%) with brain metastases in 2016 and later, 1544 (50.3%) received first-line ICI, which was associated with an improved median OS of 19.9 months (95% CI, 17.2-25.0 months; adjusted HR, 0.74; 99% CI, 0.59-0.94; *P* = .001) compared with 10.7 months with first-line non-immunotherapeutic systemic therapy (95% CI, 9.5-12.3 months) using the 78-day landmark (eTable in the Supplement; Figure 2C-D). However, among patients with liver involvement (25.3%, n = 1927; 53.0% who received first-line ICI) or elevated LDH levels (58.4%, n = 1117) a significant difference in OS between first-line ICI and first-line non-immunotherapeutic systemic therapy was not observed in multivariable analyses (liver involvement: landmark 78 days; adjusted HR, 0.77; 99% CI, 0.58-1.03; *P* = .02; elevated LDH levels: adjusted HR, 0.77; 99% CI, 0.52-1.13, *P* = .08) (eTable in the Supplement; Figure 2E-F).

### Patient, Socioeconomic, and Care Setting Features Associated With Use of First-Line Immune Checkpoint Inhibitors

From 2016 to 2019, 4330 (55.9%) patients received first-line ICI. Notably, among the 691 patients who died less than 48 days after diagnosis (ie, the median time from diagnosis to ICI initiation), 609 (88.1%) did not receive first-line ICI. Therefore, we also applied the landmark time points to the multivariable logistic regression, to help account for immortal time bias in the receipt of first-line ICI (Table). With respect to demographic factors, older patients (≥80 years) were less likely to receive first-line ICI compared with younger patients (odds ratio [OR] using the 78-day landmark = 0.42 vs ages 60 to 69 years; 99% CI, 0.31-0.57; *P* < .001), but differences in ICI use were not observed based on patients’ sex or race and ethnicity. With respect to socioeconomic factors, patients with Medicaid insurance (OR, 0.59; 99% CI, 0.41-0.86; *P* < .001) were less likely to receive first-line ICI compared with patients with private insurance. In addition, patients from zip codes with higher median household incomes were more likely to receive first-line ICI (eg, richest quartile: OR = 1.52 vs poorest quartile; 99% CI, 1.13-2.04; *P* < .001). Melanoma primary site was not associated with ICI utilization, however, metastatic site was: patients presenting with brain involvement were less likely to receive first-line ICI (OR, 0.61; 99% CI, 0.48-0.77; *P* < .001), whereas patients presenting with lung metastasis (OR, 1.46; 99% CI, 1.20-1.77; *P* < .001) or distant lymph node metastasis (OR, 1.46; 99% CI, 1.17-1.81; *P* < .001) were more likely to receive ICI. With respect to patients’ management, those who received first-line non-immunotherapeutic systemic therapy (ie, likely targeted therapy) were less likely to receive first-line ICI (single-agent: OR, 0.20; 99% CI, 0.13-0.32; *P* < .001; multiagent: OR, 0.09; 99% CI, 0.06-0.12; *P* < .001). Surgery, either of the primary site or metastases, was not associated with ICI receipt; however, patients who received first-line radiation therapy were more likely to receive first-line ICI (OR, 1.75; 99% CI, 1.38-2.21; *P* < .001). In terms of hospital practice patterns, differences were not observed by geographic location in the US, however, patients diagnosed at comprehensive community hospitals were less likely to receive first-line ICI (OR, 0.65; 99% CI, 0.52-0.81; *P* < .001) as compared with patients at academic or NCI-comprehensive cancer centers. For patients who presented after 2015 and survived at least until the 78-day landmark, the NCDB only reported the reason for a lack of first-line ICI in 210 cases, in which for 55.7% ICI was recommended but refused by the patient, for 20.0% the patient died prior to planned ICI, and for 24.3% ICI was not recommended because it was contraindicated.

**Table.** Characteristics Associated With Use of First-Line ICI in Patients With Stage IV Melanoma Diagnosed After FDA Approval^a^

Characteristic	Landmark			
	48 d (n = 4448)		78 d (n = 4154)	
	OR (99% CI)	*P* value	OR (99% CI)	*P* value
Age at diagnosis, y				
<50	1.34 (0.95-1.89)	.03	1.30 (0.91-1.85)	.06
50-59	1.03 (0.79-1.35)	.77	1.01 (0.76-1.35)	.90
60-69	1 [Reference]		1 [Reference]	
70-79	0.75 (0.58-0.96)	.003	0.79 (0.61-1.04)	.03
≥80	0.42 (0.32-0.57)	<.001	0.42 (0.31-0.57)	<.001
Sex				
Male	1 [Reference]		1 [Reference]	
Female	0.94 (0.78-1.13)	.37	0.91 (0.74-1.1)	.20
Race and ethnicity				
Asian or Pacific Islander	0.91 (0.32-2.55)	.81	0.89 (0.3-2.63)	.78
Hispanic	0.82 (0.48-1.4)	.34	0.79 (0.45-1.38)	.27
Non-Hispanic				
Black	1.77 (0.82-3.86)	.06	1.74 (0.78-3.88)	.07
White	1 [Reference]		1 [Reference]	
Other^b^	0.90 (0.39-2.08)	.75	0.76 (0.31-1.88)	.44
Unknown	0.43 (0.14-1.31)	.05	0.39 (0.13-1.21)	.03
Charlson-Deyo comorbidity index				
0	1 [Reference]		1 [Reference]	
1	0.99 (0.77-1.27)	.93	1.04 (0.8-1.36)	.71
≥2	0.76 (0.57-1)	.01	0.76 (0.56-1.03)	.02
Year of diagnosis, per y	1.13 (1.04-1.22)	<.001	1.07 (0.98-1.16)	.04
Primary skin site				
Lip	1.03 (0.04-24.54)	.98	0.95 (0.04-23.67)	.97
Eyelid	0.93 (0.03-32.69)	.96	0.89 (0.02-35.13)	.94
External ear	1.37 (0.45-4.14)	.47	1.43 (0.45-4.57)	.42
Face	0.87 (0.47-1.61)	.57	0.80 (0.42-1.51)	.36
Scalp and neck	1.07 (0.67-1.7)	.73	1.02 (0.63-1.65)	.91
Trunk	1 [Reference]		1 [Reference]	
Upper limb and shoulder	0.97 (0.63-1.51)	.87	0.96 (0.6-1.52)	.80
Lower limb and hip	1.13 (0.72-1.77)	.48	1.08 (0.68-1.72)	.67
Overlapping	1.45 (0.34-6.11)	.51	1.61 (0.34-7.69)	.43
Skin, NOS	0.91 (0.66-1.25)	.44	0.86 (0.62-1.2)	.24
Metastasis				
No	[Reference]		[Reference]	
Liver	0.99 (0.79-1.25)	.95	1.09 (0.85-1.4)	.37
Lung	1.41 (1.17-1.69)	<.001	1.46 (1.2-1.77)	<.001
Bone	1.16 (0.92-1.45)	.10	1.21 (0.95-1.55)	.04
Brain	0.56 (0.45-0.7)	<.001	0.61 (0.48-0.77)	<.001
Distant LN	1.39 (1.13-1.71)	<.001	1.46 (1.17-1.81)	<.001
Other organ	1.00 (0.83-1.2)	>.99	1.02 (0.84-1.23)	.83
Primary site surgery				
None	0.98 (0.66-1.44)	.87	1.06 (0.71-1.58)	.73
Excisional biopsy	0.84 (0.42-1.7)	.53	0.83 (0.4-1.73)	.52
Gross excision	1.00		1.00	
Wide excision/amputation	0.86 (0.57-1.29)	.33	0.84 (0.55-1.29)	.30
Radiotherapy	1.75 (1.4-2.19)	<.001	1.75 (1.38-2.21)	<.001
No	[Reference]		[Reference]	
Targeted therapy/chemotherapy				
None	1 [Reference]		1 [Reference]	
Single agent	0.22 (0.14-0.34)	<.001	0.20 (0.13-0.32)	<.001
Multiagent	0.10 (0.07-0.13)	<.001	0.09 (0.06-0.12)	<.001
Surgery of non-primary site	0.89 (0.72-1.08)	.13	0.84 (0.68-1.04)	.04
No	[Reference]		[Reference]	
Insurance status				
Not insured	0.68 (0.42-1.09)	.03	0.70 (0.42-1.15)	.06
Private insurance	1 [Reference]		1 [Reference]	
Medicaid	0.56 (0.39-0.79)	<.001	0.59 (0.41-0.86)	<.001
Medicare	0.80 (0.62-1.03)	.02	0.80 (0.62-1.05)	.03
Other Government	0.44 (0.23-0.85)	.001	0.41 (0.21-0.82)	.001
Unknown	0.52 (0.22-1.24)	.05	0.57 (0.22-1.48)	.13
Median household income of patient's zip code, $				
<38 227	1 [Reference]		1 [Reference]	
38 227-50 353	1.21 (0.91-1.61)	.09	1.30 (0.96-1.76)	.03
50 354-63 332	1.19 (0.89-1.58)	.12	1.19 (0.88-1.6)	.14
≥63 333	1.43 (1.08-1.89)	.001	1.52 (1.13-2.04)	<.001
Hospital cancer program type				
Community	0.70 (0.48-1.04)	.02	0.70 (0.46-1.05)	.02
Comprehensive community	0.66 (0.53-0.81)	<.001	0.65 (0.52-0.81)	<.001
Academic/NCI comprehensive	1 [Reference]		1 [Reference]	
Integrated Network	0.77 (0.6-0.99)	.007	0.75 (0.58-0.98)	.006
Hospital location				
New England	1.35 (0.82-2.22)	.12	1.32 (0.78-2.25)	.17
Middle Atlantic	1.00 (0.72-1.39)	.98	1.01 (0.72-1.43)	.94
South Atlantic	0.90 (0.68-1.19)	.32	0.91 (0.67-1.22)	.39
Central				
East North	1 [Reference]		1 [Reference]	
East South	0.86 (0.59-1.25)	.29	0.95 (0.63-1.43)	.75
West North	1.39 (0.94-2.06)	.03	1.47 (0.97-2.22)	.02
West South	0.92 (0.63-1.33)	.55	0.87 (0.59-1.29)	.36
Mountain	1.11 (0.73-1.69)	.52	1.18 (0.76-1.84)	.33
Pacific	0.98 (0.71-1.36)	.89	0.97 (0.69-1.36)	.81

Abbreviations: ICI, immune checkpoint inhibitor; LN, lymph node; OR, odds ratio.

^a^ Table data calculated using multivariable logistic regression.

^b^ Other race and ethnicity was defined based on what the US National Cancer Database (NCDB) reported; NCDB race reported either as other (code 98) or American Indian, Aleutian, or Eskimo (code 03).

## Discussion

In this national analysis, we found that use of ICI therapy in the first-line setting for stage IV melanoma has increased substantially in United States, although first-line ICI use continued to lag even after FDA approval in late 2015—particularly for patients who were older, had brain involvement, Medicaid insurance, or were from the lowest quartile of households by zip-code level income. Notably, from 2010 to 2019, the median OS experienced by patients with stage IV melanoma in the US has more than doubled, to an estimated 17.5 months. Following FDA approval, we found first-line ICI use to be independently associated with substantial improvements in OS for patients with stage IV melanoma, including among those with brain metastases. Given the high mortality rate experienced by patients with stage IV melanoma, we incorporated landmark time points in all of our analyses to help account for the immortal time bias associated with the receipt of ICI using both the median and 75th percentile of time from diagnosis to ICI initiation (ie, survival time bias, in which patients who received ICI would be biased toward improved survival because by definition they would not be able to experience the end point of death between diagnosis and ICI receipt, whereas patients who did not receive ICI may have experienced an early mortality prior to receiving planned ICI).

Following the publication of a phase 3 study that demonstrated an improvement in OS among patients with metastatic melanoma treated with ipilimumab in the second-line setting,^16^ Robert et al^17^ assessed the efficacy of ipilimumab compared with standard chemotherapy in the first-line setting for patients with metastatic melanoma and demonstrated significantly improved OS even among previously untreated patients (median survival: 11.2 vs 9.1 months with ipilimumab-dacarbazine vs dacarbazine alone). Subsequently, the KEYNOTE-006 and Checkmate-067 studies demonstrated that pembrolizumab or nivolumab monotherapy improved OS compared to monotherapy with ipilimumab.^18,19^ Checkmate-067 and 069 also demonstrated improved response rates with combination therapy (nivolumab and ipilimumab) compared with ipilimumab monotherapy.^10,19^ Collectively, these studies led to NCCN recommendations (and FDA approvals) since late 2015 for either anti–PD-1 monotherapy with nivolumab or pembrolizumab or combination anti–PD-1 and anti–CTLA-4 therapy with nivolumab andipilimumab, as the preferred first-line systemic treatments for patients with stage IV melanoma.^3^ In line with these trials’ efficacy results, we found that first-line ICI after FDA approval was associated with an almost doubling of the median OS compared with patients who received first-line targeted therapy and (ie, 44 months vs 16 months among those patients who survived at least until 78 days), and an increase in the 3-year OS rate to 53% from 36%.

Moreover, it should be noted that among patients with *BRAFV600*-variant metastatic melanoma, targeted BRAF inhibitors and MEK inhibitors have also demonstrated antitumor efficacy and overall survival benefit, the optimal sequencing of ICI vs BRAF and MEK inhibitors remains unclear.^20^ The recent DREAMseq trial (ECOG-ACRIN EA6134) randomized treatment-naive patients with *BRAFV600*-variant metastatic melanoma to receive initial ICI or initial *BRAF-* and *MEK*-directed therapy, followed by the opposite treatment at the time of disease progression.^21^ Overall, they found that even among this population of patients with targetable variations, beginning treatment with ICI resulted in a superior overall survival and a longer duration of response than if treatment was started with targeted therapy. Our findings suggest that patients in the US who received single-agent or multiagent targeted therapy or chemotherapy in the front-line setting are less likely to also receive first-line ICI; however, the results of the DREAMseq trial underscore the need for strong consideration for ICI in the first-line management of patients with metastatic melanoma, including those with targetable variations.

We further evaluated the OS associated with first-line ICI for several subpopulations that were historically challenging to treat. For instance, the practice-changing studies previously described that led to the recommendations for anti–PD-1 ICI in the first-line setting for patients with metastatic melanoma disproportionately excluded patients with brain metastases. More specifically, only 9% of patients in KEYNOTE-006 and 3% of patients in Checkmate-067 and -069 had brain metastases, and subanalyses pertaining to just patients with intracranial disease were not reported, thereby limiting the applicability of these efficacy data to patients with intracranial disease.^10,18,19^ There has been subsequent work among patients with melanoma brain metastases to better understand the efficacy of ICI among such patients, albeit limited by the small number of patients in these studies, which has been extensively reviewed elsewhere.^8,22,23,24,25,26^ Together, the clinical trials of ICI in melanoma brain metastases illustrated acceptable toxicity, favorable intracranial response rates, and OS improvements. Based on the findings from CheckMate-204, combination nivolumab and ipilimumab is now the standard of care for management of brain metastases.^26^ We previously demonstrated the OS benefits experienced by patients with melanoma brain metastasis in the US largely in the ipilimumab monotherapy era—results that were extended and confirmed by others.^15,27,28^ Herein, we found that ICI following FDA approval of first-line anti–PD-1 was also associated with extensive OS benefit in patients with intracranial metastases from melanoma, improving median OS to an estimated 20 months among patients who survived at least 78 days. However, first-line ICI use remained disproportionately lower even among patients with brain metastases diagnosed in 2016 to 2019, underscoring persistent gaps in the incorporation of ICI into the management of these patients, although ICI use has likely improved markedly in the years since, given that ChekMate-204’s positive findings with nivolumab and ipilimumab were published in the latter half of 2018. The NCDB unfortunately does not permit distinguishing between anti–PD-1 monotherapy and anti–PD-1/anti–CTLA4 combination therapy.

As a secondary aim, we evaluated the factors associated with underutilization of first-line ICI following the publication of NCCN recommendations and FDA approval for front-line anti–PD-1 in late 2015. We and others have previously analyzed ICI use patterns for patients with advanced melanoma primarily in the ipilimumab era, when anti–PD-1 use was likely restricted to clinical trial or off-label use.^2,12,29,30,31^ Herein we found that first-line ICI use has increased to 63% of newly diagnosed patients with stage IV melanoma in 2019, from 9% in 2010 and 39% in 2015 (the year leading up to FDA approval of first-line anti–PD-1). In order to investigate the patient, socioeconomic, and care setting factors associated with underutilization of first-line ICI, we also incorporated landmark time points into our analysis to help account for the immortal time bias associated with receipt of ICI, in which early mortality would preclude patients who would otherwise receive first-line ICI from doing so. Following FDA approval of anti–PD-1, we found that first-line ICI was underutilized among older patients and patients with brain metastases, although this has likely improved since the data from CheckMate-204 were reported. Furthermore, patients with Medicaid insurance or from the poorest zip codes (by median household income) were less likely to receive ICI, suggesting that some financial barriers may hinder access to first-line ICI. Finally, although improvements have been made across cancer program times in ICI use, comprehensive community cancer programs continued to have disproportionately lower use of ICI in the first-line setting compared with academic or NCI-designated comprehensive cancer centers.

### Limitations

This study has some limitations. First, the NCDB does not report details regarding the specific agents, doses, schedules, duration of treatment, or associated toxicities for immunotherapy or targeted therapy or chemotherapy; nor does the NCDB report treatments used beyond the first-line setting. Consequently, we could not distinguish anti–PD-1 monotherapy from combined anti–PD-1 and anti–CTLA-4. Second, the NCDB lacked details regarding localized treatment of metastases, which play a crucial role in many melanoma treatment settings, especially brain metastases.^11,32,33,34,35,36^ For example, data regarding limited (ie, resectable) vs distant (ie, usually unresectable) metastatic disease were not granularly reported by the NCBD; however, we included resection of a nonprimary lesion in our multivariable analyses to help account for resectability. The NCDB additionally does not report the use of corticosteroids—an especially pertinent consideration in the brain metastasis setting, which increasingly has been shown to compromise the efficacy of ICI.^37,38,39,40^ Third, the NCDB does not report data relating to progression-free survival or objective response assessment.

## Conclusions

In this national-level cohort study, we found a substantial increase in the use of ICI in the first-line setting for patients with metastatic melanoma, associated with improvement in their OS. These benefits were also seen in patients presenting with brain involvement. However, despite the steady increase in use over the last decade, an estimated 38% of patients diagnosed with metastatic melanoma in the US still did not receive ICI in the first-line setting in 2019—with use lagging for older patients and patient with brain metastasis, but also varying by patients’ socioeconomic statuses and care settings—indicating a need for ongoing efforts to increase the use of these important therapies.

## Notes

Supplement. **eTable.** Association Between First-Line ICI and OS in Stage IV Melanoma Patients Diagnosed Following FDA Approval, Using Multivariable Cox Regression
