# Supplementary material for: Use of First-Line Immune Checkpoint Inhibitors and Association With Overall Survival Among Patients With Metastatic Melanoma in the Anti–PD-1 Era
Source: JAMA Netw Open. 2022 Aug 25;5(8):e2225459. doi: 10.1001/jamanetworkopen.2022.25459 (PMC9412220; doi:10.1001/jamanetworkopen.2022.25459)
Supplement: Supplement. — eTable. Association Between First-Line ICI and OS in Stage IV Melanoma Patients Diagnosed Following FDA Approval, Using Multivariable Cox Regression [file jamanetwopen-e2225459-s001.pdf]

Supplemental Online Content

Lamba N, Ott PA, Iorgulescu JB. Use of First-Line Immune Checkpoint Inhibitors and Association With Overall Survival Among Patients With Metastatic Melanoma in the Anti-PD-1 Era. *JAMA Netw Open.* 2022;5(8):e2225459. doi:10.1001/jamanetworkopen.2022.25459

eTable. Association Between First-Line ICI and OS in Stage IV Melanoma Patients Diagnosed Following FDA Approval, Using Multivariable Cox Regression

This supplemental material has been provided by the authors to give readers additional information about their work.

© 2022 Lamba N et al. *JAMA Network Open*.

eTable. Association between first-line ICI and OS in stage IV melanoma patients diagnosed following FDA approval, using multivariable Cox regression

	All						Brain metastases						Liver metastases						High LDH					
**Landmark timepoint**	**Landmark 48 days**			**Landmark 78 days**			**Landmark 48 days**			**Landmark 78 days**			**Landmark 48 days**			**Landmark 78 days**			**Landmark 48 days**			**Landmark 78 days**		
**n=**		3,408			3,239			1,251			1,151			802			725			725			488	
	**HR**	**99% CI**	**p val**	**HR**	**99% CI**	**p val**	**HR**	**99% CI**	**p val**	**HR**	**99% CI**	**p val**	**HR**	**99% CI**	**p val**	**HR**	**99% CI**	**p val**	**HR**	**99% CI**	**p val**	**HR**	**99% CI**	**p val**
**1st-line systemic therapy**																								
Targeted/chemotherapy	Ref			Ref			Ref			Ref			Ref			Ref			Ref			Ref		
ICI	0.76	(0.65-0.88)	**<0.001**	0.72	(0.62-0.85)	**<0.001**	0.78	(0.63-0.97)	**0.003**	0.74	(0.59-0.94)	**0.001**	0.82	(0.63-1.08)	0.06	0.77	(0.58-1.03)	0.019	0.78	(0.54-1.12)	0.07	0.77	(0.52-1.13)	0.08
**Age at diagnosis, yr**																								
<50	0.92	(0.76-1.11)		0.93	(0.76-1.12)		0.85	(0.65-1.11)		0.82	(0.62-1.09)		1.03	(0.74-1.43)		1.05	(0.74-1.49)		1.30	(0.85-1.98)		1.37	(0.88-2.14)	
50-59	0.94	(0.78-1.13)		0.89	(0.73-1.09)		0.94	(0.72-1.23)		0.89	(0.67-1.18)		0.91	(0.65-1.28)		0.84	(0.57-1.22)		1.20	(0.76-1.87)		1.03	(0.63-1.69)	
60-69	Ref			Ref			Ref			Ref			Ref			Ref			Ref			Ref		
70-79	1.32	(1.1-1.57)		1.29	(1.07-1.56)		1.25	(0.95-1.63)		1.19	(0.89-1.58)		1.41	(1.01-1.96)		1.44	(1.01-2.06)		1.27	(0.78-2.06)		1.30	(0.78-2.17)	
≥80	1.60	(1.29-1.98)		1.55	(1.24-1.94)		1.28	(0.88-1.88)		1.13	(0.74-1.73)		1.29	(0.86-1.94)		1.35	(0.87-2.09)		1.72	(0.99-2.97)		1.65	(0.91-2.98)	
**Charlson-Deyo comorbidity index**																								
0	Ref			Ref			Ref			Ref			Ref			Ref			Ref			Ref		
1	1.06	(0.88-1.27)		1.09	(0.9-1.32)		1.00	(0.76-1.31)		1.04	(0.78-1.39)		0.98	(0.69-1.39)		0.98	(0.68-1.43)		0.88	(0.56-1.38)		0.91	(0.56-1.47)	
≥2	1.40	(1.13-1.73)		1.38	(1.1-1.74)		1.18	(0.85-1.64)		1.18	(0.83-1.67)		1.23	(0.79-1.9)		1.14	(0.7-1.86)		1.07	(0.58-1.99)		1.07	(0.55-2.1)	
**Radiotherapy**																								
No	Ref			Ref			Ref			Ref			Ref			Ref			Ref			Ref		
Yes	1.32	(1.13-1.55)		1.32	(1.12-1.55)		1.35	(1.05-1.73)		1.36	(1.04-1.77)		1.31	(1-1.72)		1.26	(0.94-1.69)		1.36	(0.95-1.94)		1.31	(0.89-1.92)	
**Year of diagnosis, per yr**	0.95	(0.87-1.02)		0.94	(0.87-1.03)		1.00	(0.89-1.13)		1.01	(0.89-1.15)		0.95	(0.82-1.1)		0.96	(0.82-1.12)		1.02	(0.85-1.23)		0.99	(0.81-1.21)	
**Surgery of non-primary site**																								
No	Ref			Ref			Ref			Ref			Ref			Ref			Ref			Ref		
Yes	0.64	(0.55-0.75)		0.68	(0.58-0.79)		0.62	(0.5-0.77)		0.67	(0.54-0.84)		0.71	(0.51-1)		0.76	(0.53-1.08)		0.53	(0.35-0.81)		0.56	(0.36-0.87)	
**Brain metastases**																								
No	Ref			Ref									Ref			Ref			Ref			Ref		
Yes	1.69	(1.44-1.98)		1.63	(1.38-1.92)		All have brain mets			All have brain mets			1.43	(1.08-1.89)		1.40	(1.04-1.9)		1.14	(0.8-1.63)		1.14	(0.78-1.66)	
**Lung metastases**																								
No	Ref			Ref			Ref			Ref			Ref			Ref			Ref			Ref		
Yes	1.10	(0.97-1.25)		1.06	(0.93-1.21)		1.19	(0.98-1.45)		1.15	(0.93-1.42)		1.05	(0.82-1.34)		0.97	(0.74-1.26)		1.22	(0.89-1.68)		1.14	(0.81-1.59)	
**Other organ metastases**																								
No	Ref			Ref			Ref			Ref			Ref			Ref			Ref			Ref		
Yes	1.08	(0.95-1.23)		1.03	(0.9-1.18)		1.01	(0.82-1.23)		0.97	(0.78-1.2)		0.99	(0.78-1.25)		0.92	(0.71-1.2)		1.08	(0.8-1.46)		1.01	(0.73-1.4)	
**Distant LN metastases**																								
No	Ref			Ref			Ref			Ref			Ref			Ref			Ref			Ref		
Yes	1.10	(0.96-1.26)		1.11	(0.96-1.28)		1.08	(0.87-1.35)		1.09	(0.86-1.38)		0.99	(0.78-1.27)		0.99	(0.76-1.29)		1.11	(0.81-1.51)		1.12	(0.8-1.57)	
**Liver metastases**																								
No	Ref			Ref			Ref			Ref									Ref			Ref		
Yes	1.50	(1.3-1.73)		1.44	(1.24-1.68)		1.35	(1.08-1.7)		1.31	(1.02-1.68)		All have liver mets			All have liver mets			1.19	(0.86-1.64)		1.15	(0.81-1.62)	
**Bone metastases**																								
No	Ref			Ref			Ref			Ref			Ref			Ref			Ref			Ref		
Yes	1.57	(1.36-1.81)		1.57	(1.35-1.83)		1.37	(1.1-1.72)		1.38	(1.08-1.76)		1.49	(1.18-1.88)		1.50	(1.16-1.93)		1.57	(1.15-2.15)		1.55	(1.11-2.17)	

ICI = immune checkpoint inhibitor, HR = hazard ratio, CI = confidence interval, LN = lymph node

P values were only calculated and displayed for the primary association of interest, that between first-line ICI and OS. 99% CIs are reported for all other associations.

© 2022 Lamba N et al. *JAMA Network Open.*
